# New Delhi Metallo-β-Lactamase-5–Producing *Escherichia coli* in Companion Animals, United States

**DOI:** 10.3201/eid2602.191221

**Published:** 2020-02

**Authors:** Stephen D. Cole, Laura Peak, Gregory H. Tyson, Renate Reimschuessel, Olgica Ceric, Shelley C. Rankin

**Affiliations:** University of Pennsylvania School of Veterinary Medicine, Philadelphia, Pennsylvania, USA (S.D. Cole, S.C. Rankin);; Louisiana State University, Baton Rouge, Louisiana, USA (L. Peak);; US Food and Drug Administration, Silver Spring, Maryland, USA (G.H. Tyson, R. Reimschuessel, O. Ceric)

**Keywords:** New Delhi metallo-β-lactamase-5, Escherichia coli, bacteria, antimicrobial resistance, carbapenem-resistant Enterobacteriaceae, pets, companion animals. dogs, cats, veterinary medicine, One Health, public health, United States

## Abstract

We report isolation of a New Delhi metallo-β-lactamase-5–producing carbapenem-resistant *Escherichia coli* sequence type 167 from companion animals in the United States. Reports of carbapenem-resistant *Enterobacteriaceae* in companion animals are rare. We describe a unique cluster of *bla*_NDM-5_–producing *E. coli* in a veterinary hospital.

Carbapenems are critically useful antimicrobial drugs that are reserved for treatment of infections caused by multidrug-resistant, gram-negative bacteria (*1*). Carbapenem-resistant *Enterobacteriaceae* (CRE) have emerged as a major cause of human healthcare-associated infections and are a major clinical and public health problem (*1*). The most common mechanism of resistance is production of carbapenemases, which hydrolyze carbapenems and many other β-lactams. The genes that encode carbapenemases are found on conjugative plasmids and commonly fall in the following classes: KPC (*Klebsiella pneumoniae* carbapenemase), NDM (New Delhi metallo-β-lactamase), IMP (imipenamase), and VIM (Verona integron-encoded metallo-β-lactamase) (*1*). Control of these infections in human healthcare settings is a challenge because the organisms colonize the gastrointestinal tract and can go undetected (*1*). Reports of CRE in animals and animal settings are rare but have been documented in livestock, wildlife, and companion animals (*2*).

In April 2019, passive surveillance by the Veterinary Laboratory Information and Response Network of the US Food and Drug Administration identified the *bla*_NDM-5_ gene in a carbapenem-resistant *Escherichia coli* isolated from a dog in July 2018. This isolate belonged to sequence type 167 (ST167).

A retrospective review of hospital records showed that, during July 11–August 3, 2018, seven carbapenem-resistant *E. coli* isolates were isolated from 6 animals (Table). Isolates were obtained from 5 dogs and 1 cat; all were from respiratory tract specimens, except for 2 isolates from the urine of 1 dog. All animals were housed in the intensive care unit for >24 hours (Appendix Figure). All animals overlapped with >1 other affected animal. 

**Table:** Clinical characteristics of New Delhi metallo-β-lactamase-5–producing *Escherichia coli* isolates from 6 companion animals, United States, 2018*

Isolate ID	BioSample accession no.	Source	Species	Breed	Age, y/sex	Concurrent diagnosis	Antimicrobial drugs in the past 30 days	Outcome
24213-18	SAMN11230749	Endotracheal wash	Canine	Great Dane	11/F	Megaesophagus; aspiration pneumonia	AMP, CLI, CAZ, ENR, MTZ	Discharged
24920-18	SAMN12190106	Lung tissue	Canine	Rottweiler	8/M	Pheochormocytoma; aspiration pneumonia	AMP, AZM, ENR, MTZ	Discharged
27025-18-1, 27025-18-2	SAMN12189820, SAMN12190134	Urine (cystocentesis)	Canine	Mixed breed	13/F	Atrioventricular block; acute kidney injury	AMP, AZM, CFZ, ENR, MTZ	Discharged
27241-18	SAMN12190501	Endotracheal wash	Canine	Beagle	5/M	Septic peritonitis after foreign body removal; protein-losing nephropathy; suspected pulmonary thromobembolism	AMP, AZM, FOX, PTZ, MTZ	Euthanized
27609-18	SAMN12190410	Endotracheal wash	Feline	Domestic shorthair	8/F	Asthma	AZM	Discharged
27614-18	SAMN12190436	Endotracheal wash	Canine	Standard poodle	8/F	Laryngeal paralysis; pneumonia	AZM, AMC, ENR	Discharged

*All animals had been spayed or castrated. AMC, amoxicillin/clavulanate; AMP, ampicillin; AZM, azithromycin; CAZ, ceftazidime; CLI, clindamycin; CFZ, cefazolin; ENR, enrofloxacin; FOX, cefoxitin; ID, identification; MTZ, metronidazole; NCBI, National Center for Biotechnology Information; PTZ, piperacillin/tazobactam.

We evaluated antimicrobial use; 5/6 animals received >4 antimicrobial drugs before specimen submission. No animals received a carbapenem drug in the 30-day period before sample submission. A β-lactam was given to 5/6 animals, azithromycin to 5/6 animals, metronidazole to 4/6 animals, and enrofloxacin to 4/6 animals (Table).

The first isolate, *E. coli* 24213-18, was sequenced by using a Pacific Biosciences Sequel Sequencer (https://www.pacb.com) and uploaded to GenBank (BioSample SAMN11230749). This testing confirmed *E. coli* ST167 and identified a circular IncFII plasmid of 139,547 bp, which contained the *bla*_NDM-5_ gene and additional resistance genes: *tet(A)*, *aac(6′)-Ib-cr*, *aadA5*, *aadA2*, *bla*_OXA-1_, *bla*_CTX-M-15_, *catB3*, *dfrA17*, *dfrA12*, *sul1* (2 copies), and *mph(A)* (*3*).

Whole-genome sequencing was performed on all 7 isolates (24213-18, 24920-18, 27025-1-18, 27025-2-18, 27241-18, 27609-18, and 27614-18) by using the Illumina MiSeq platform (https://www.illumina.com). We identified antimicrobial resistance genes by using the National Center for Biotechnology Information Pathogen Detection Isolates Browser, which uses AMRFinder (https://www.ncbi.nlm.nih.gov/pathogens/antimicrobial-resistance/AMRFinder). The following genes were found in all 7 isolates: *aac 3-IId*, *aac (6′)-Ib-cr5*, *aadA2*, *aadA22*, *aadA5*, *bla*_CTX-M-15_, *bla*_NDM-5_, *bla*_OXA-1_, *bla*_TEM-1_, *ble*, *catB3*, *dfrA12*, *dfrA17*, *mph(A)*, *qacEdelta1*, *sul1*, and *tet(A)*. The *floR* gene was detected in all isolates except 27025–1-18. PlasmidFinder (https://cge.cbs.dtu.dk/services/PlasmidFinder) analysis confirmed the presence of an IncFII plasmid in all isolates.

NDM-5–producing *E. coli* have been reported in dogs from Finland, South Korea, and Algeria (*3**–**5*). The isolates from Finland were also ST167; the isolates from South Korea and Algeria were obtained from canine feces and identified as ST410 and ST1284. ST1284 is a double-locus variant of ST167, which suggests possible distant relatedness of these isolates; ST410 does not share any multilocus sequence type alleles with ST167 (*5*). In 2011, the NDM-5 carbapenemase was described in an isolate of *E. coli* (ST648) from a human in the United Kingdom who was previously hospitalized in Goa, India (*6*). In February 2018, three isolates of NDM-5–positive *E. coli* (ST43) were isolated from 2 patients in a skilled nursing facility in New York, New York (*7*). Spread of NDM-5–positive *E. coli* has occurred globally and included reports of ST167 in persons in Europe and Asia (*8*,*9*).

Healthcare-associated spread of this *E. coli* strain in the veterinary intensive care unit emphasizes the need to rapidly identify and characterize carbapenem-resistant isolates from animals. Methods to control the spread of CRE in veterinary medical settings have not yet been studied; these studies are needed to limit the spread of these pathogens in animal populations. Control measures in human healthcare settings include strict hand hygiene, use of personal protective equipment, and environmental decontamination (*10*). The risk for transmission of CRE from animals to persons is currently poorly understood. 

It has been documented that *bla*_NDM-5_, ST167, and carbapenem-resistant *E. coli* strains can infect humans and animals (*4*). Additional investigations are needed in the context of transmission between humans and animals. Characterization of CRE isolates from animals is needed to build a knowledge base and provide guidance for future studies because CRE will continue to emerge in veterinary medical settings. CRE will be a major challenge across all health fields as these organisms become more prevalent in the community. A One Health approach to antimicrobial resistance surveillance, infection prevention, and antimicrobial stewardship could limit the spread and potential global dominance of CRE.

Appendix Additional information on New Delhi metallo-β-lactamase-5–producing *Escherichia coli* in companion animals, United States.
