# Supplementary material for: New Delhi Metallo-β-Lactamase-5–Producing Escherichia coli in Companion Animals, United States
Source: Emerg Infect Dis. 2020 Feb;26(2):381–3. doi: 10.3201/eid2602.191221 (PMC6986821; doi:10.3201/eid2602.191221)
Supplement: Appendix — Additional information on New Delhi metallo-β-lactamase-5–producing Escherichia coli in companion animals, United States. [file 19-1221-Techapp-s1.pdf]

Article DOI: https://doi.org/10.3201/eid2602.191221

New Delhi Metallo-β-Lactamase-5–Producing *Escherichia coli* in Companion Animals, United States

Appendix

Animal / Day	0	1	2	3	4	5	6	7	8	9	10	11	12	13	14	15	16	17	18	19	20	21	22	23	24	25	26	27	28	29	30	31
24213-18	A	X	X	X	X	X	X	X	X*	X	X	X	X	X	D				O						O							
24920-18	A	X	X	X	X	X	X	X	X	X	X*	X	X	X	H	D																
27025-18														A	X	X	X	X	X	X	D		O					O*				
27241-18																					A	X	X	X	X	H	D		A	X*	D	
27609-18														A	X	X	X	X	X	D						O					O	O*
27614-18																														A	X	H*

Appendix Figure. Time line characteristics of 6 companion animals infected with New Delhi metallo-β-lactamase-5–producing *Escherichia coli*, United States, 2018. A, admitted to hospital; D, discharged from hospital; H, hospitalized but not in intensive care unit; O, outpatient visit; X, hospitalized in intensive care unit; *, culture submission date.
